# A CRISPR-Cas12a-Responsive DNA Tetrahedron

**DOI:** 10.1021/acs.biochem.6c00589

**Published:** 2026-09-16

**Authors:** Shubhajit Singha, Mehmet V. Yigit, Arun Richard Chandrasekaran

**Affiliations:** † Department of Chemistry, 1084 University at Albany, State University of New York, Albany, New York 12222, United States; ‡ The RNA Institute, University at Albany, State University of New York, Albany, New York 12222, United States; § Department of Nanoscale Science and Engineering, 1084 University at Albany, State University of New York, Albany, New York 12222, United States

## Abstract

Functionalizing DNA nanostructures for stimuli-responsive reconfiguration has been a key area of development in DNA nanotechnology. Here, we demonstrate programmable control over the structural integrity of a tetrahedral DNA nanostructure using an external DNA stimulus that is modulated through an intermediate CRISPR-Cas12a reaction. We designed toehold-containing DNA tetrahedra that can be disassembled by a DNA or RNA invading strand. Activation of the Cas12a enzyme by double-stranded DNA provides precise control over the disassembly of the DNA tetrahedra by digesting the DNA invading strand but not the RNA, enabling highly responsive and programmable DNA architectures. Further, targeted activation of Cas12a by conserved genomic regions of three bacterial pathogens, namely *Listeria monocytogenes*, *Campylobacter jejuni*, and Shiga toxin-producing *Escherichia coli*, provides biologically relevant responsiveness and demonstrates the platform’s potential for future applications. Our results show that the DNA tetrahedra are observed in the presence of the genomic target but not observed in its absence due to disassembly, demonstrating a highly programmable and responsive molecular level architecture.

## Graphical abstract

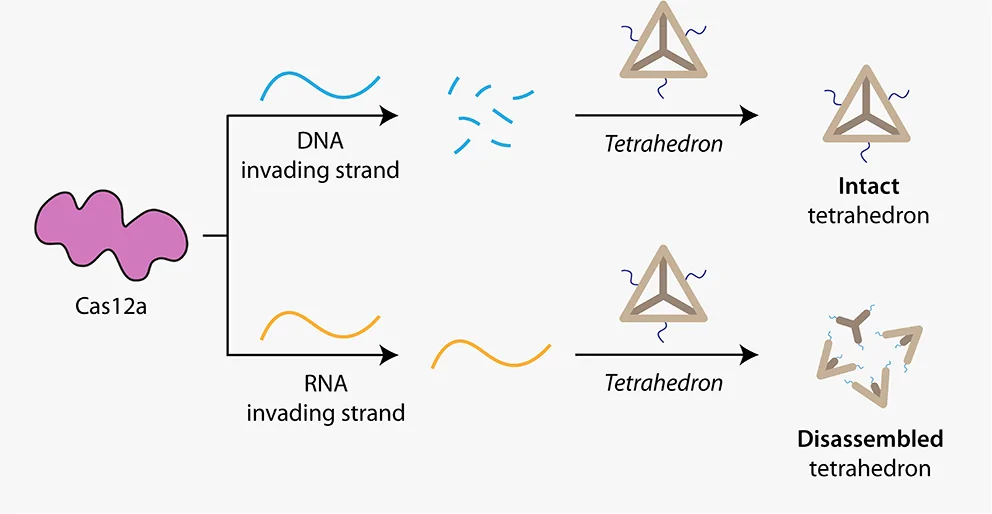

DNA nanotechnology allows the construction of well-defined nanoscale structures using DNA base-pairing.^1^ This programmability, in addition to the nanoscale features of DNA and the ability to synthesize chemically modified DNA, has enabled precision in the design and assembly of complex yet functional DNA nanostructures. Over the years, both static and dynamic DNA nanostructures have been developed for applications such as biosensing,^2,3^ drug delivery,^4,5^ and bioimaging.^6,7^ Among these, dynamic DNA nanostructures are especially of interest because of the ability to change their conformation in response to an external stimulus. One of the most used methods to control such changes is toehold-mediated strand displacement. In this process, an invading oligonucleotide binds to a short single-stranded region called a toehold and replaces an existing strand, leading to structural reconfiguration.^8,9^ Because of its versatility, this approach has been widely used in DNA circuits,^10,11^ logic systems,^12,13^ and reconfigurable nanostructures.^14,15^ Several methods have been developed to control strand displacement, including toehold length,^16^ the introduction of mismatches,^17^ and the use of external triggers such as light,^18^ pH,^19^ or enzymes.^18,20^

In our previous work, we showed that strand displacement in a DNA tetrahedron can be inhibited by removing the toehold.^18^ In that work, the DNA tetrahedron was disassembled using an invading strand, but once the toehold was removed, disassembly was inhibited even when the invading strand was present. This showed that the toehold plays a key role in controlling the disassembly. Another approach to regulate DNA nanostructure disassembly is by controlling the availability of the invading strand. Instead of making any changes to the building blocks of the DNA nanostructure (i.e., the toehold), the structural reconfiguration is regulated by external stimuli under defined conditions. CRISPR Cas12a provides a useful way to achieve such precise and programmable control over the external stimuli. Upon recognition of a double stranded (ds) DNA target, Cas12a is activated and cleaves any single stranded DNA in its vicinity.^21–24^ Since Cas12a cleaves DNA but not RNA, DNA and RNA invading strands are perceived differently by the DNA nanostructures. Here, we used three different conserved genomic regions of bacterial pathogens to activate Cas12a which in turn regulates DNA tetrahedron disassembly through a DNA invading strand. This combinatorial approach, where CRISPR-Cas12a programmed to recognize specific genomic target sequences controls the stimulus that is responsible for DNA nanostructure reconfiguration, provides a unique strategy to link structural changes in DNA nanostructures with nucleic acid recognition.

To demonstrate our strategy, we chose to use the DNA tetrahedron assembled from 3-point-star motifs (Figure 1a).^25^ Each 3-point-star motif consists of a long strand (Strand L), three copies of a medium strand (Strand M), and three copies of a short strand (Strand S). Four 3-point-star motifs connect via double sticky ends on each arm to form a DNA tetrahedron. To allow DNA tetrahedron disassembly using strand displacement, we used our prior knowledge^18^ to include an 8 nt overhang on strand S that results in the assembled DNA tetrahedron containing two toeholds per edge (Figure S1 and Table S1). An invading strand complementary to the toehold and to the rest of strand S binds to the toehold and initiates branch migration, displacing strand S from the tetrahedron. Removal of strand S disrupts the sticky end interactions between adjacent motifs, leading to tetrahedron disassembly (Figure 1b). We first demonstrated the assembly of the tetrahedron and the disassembly in the presence of a DNA or RNA invading strand using native polyacrylamide gel electrophoresis (PAGE). The disappearance of the tetrahedron band confirms the disassembly of the structure (Figure 1c, lanes 3 and 4).

**Figure 1:**
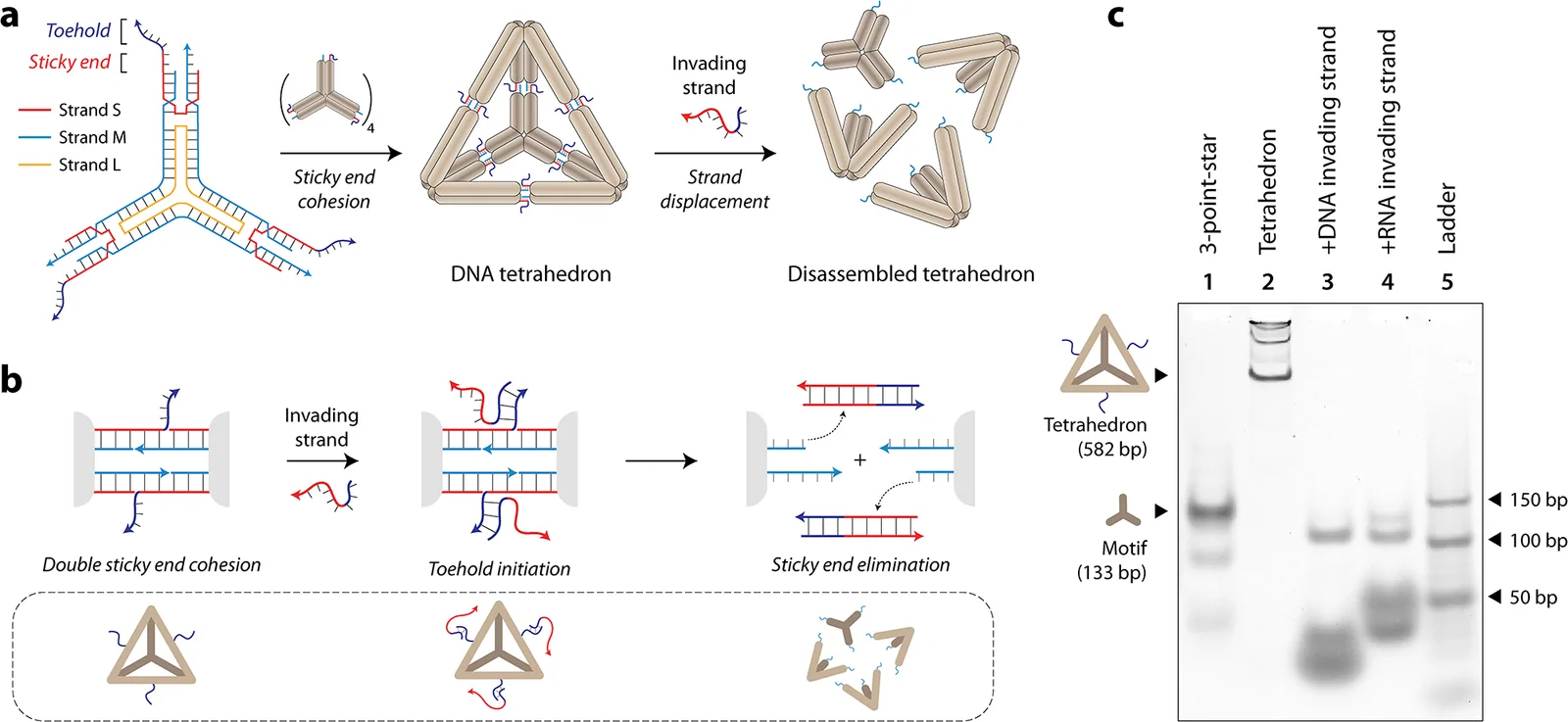
DNA tetrahedron assembly and disassembly. (a) Schematic illustration of DNA tetrahedron assembly from three-point-star motifs and disassembly by strand displacement. (b) Binding of the invading strand to the toeholds of the DNA tetrahedron edge resulting in the elimination of sticky ends and tetrahedron disassembly. (c) Non-denaturing gel showing the assembly and disassembly of DNA tetrahedra.

Since the invading strand is essential for disassembly, we regulated its availability using CRISPR-Cas12a, an RNA-guided DNA endonuclease that is activated in the presence of a model DNA target. The CRISPR-Cas12a was activated through a 50-nt long synthetic dsDNA identified from the conserved genomic regions of *Listeria monocytogenes* (Target_L_) and corresponding crRNA (crRNA_L_) designed for this target (Table S2). Upon recognition of Target_L_, activated Cas12a degrades the DNA invading strand, preventing strand displacement and retaining the intact tetrahedron, whereas the RNA invading strand remains unaffected due to its resistance to Cas12a-cleavege and induces tetrahedron disassembly (Figure 2a). We first optimized the digestion of the DNA invading strand (1 μM) with varying concentrations of active Cas12a at 37 °C for 1 h, observing efficient digestion at 125 nM Cas12a (Figure 2b and Figure S2a). We further examined the effect of incubation time and found that digestion was complete within 64 min (Figure 2c and Figure S2b). Based on these results, we chose 125 nM CRISPR-Cas12a concentration and 1 h incubation for further experiments.

**Figure 2:**
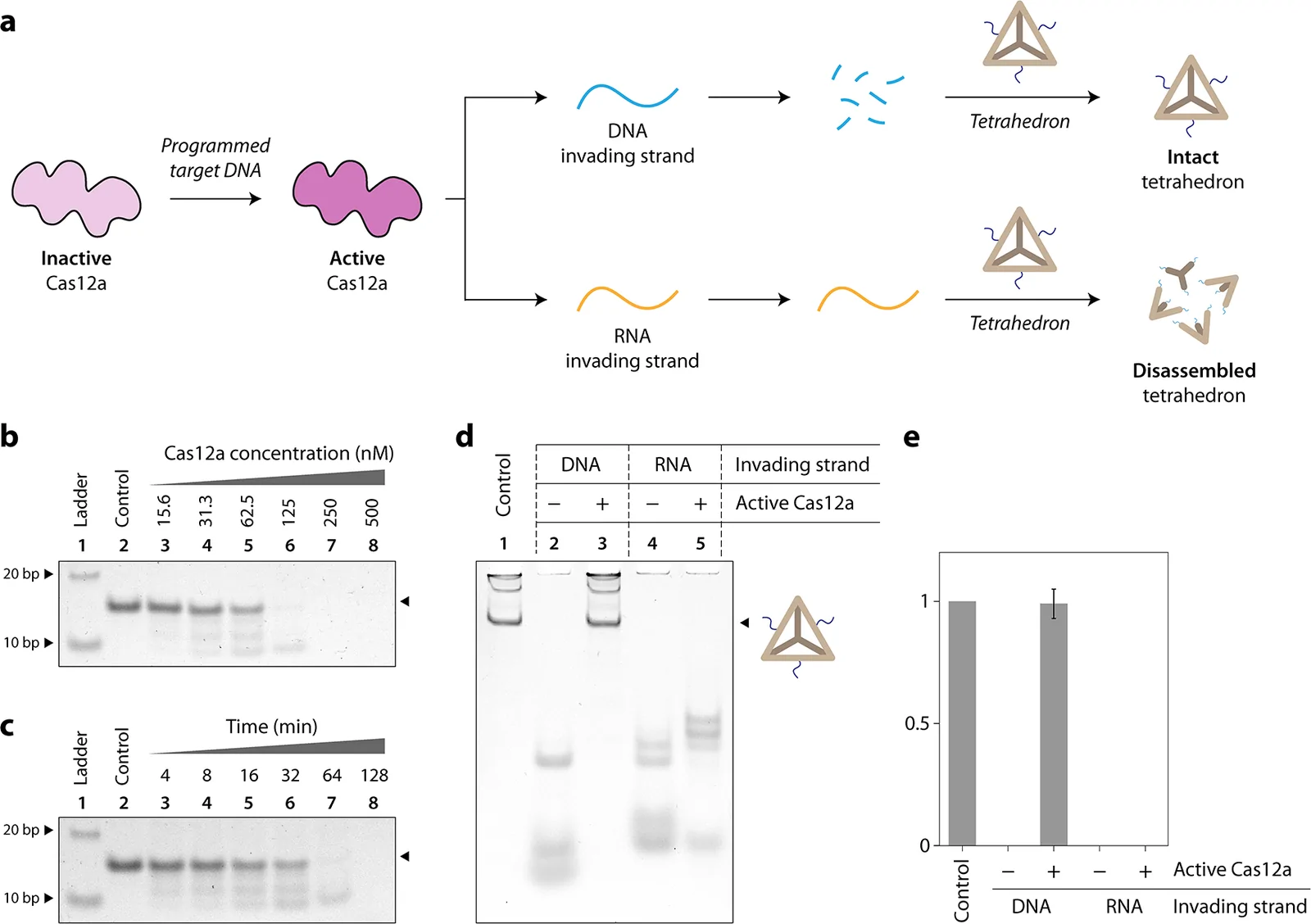
CRISPR-Cas12a triggered DNA tetrahedra disassembly. (a) Active Cas12a degrades a DNA invading strand but not an RNA invading strand. In the presence of a digested DNA invading strand, the tetrahedron remains intact. In the presence of an intact RNA invading strand, the tetrahedron is disassembled due to toehold displacement. Optimization of (b) Cas12a concentration and (c) incubation time with the DNA invading strand. (d, e) Non-denaturing gel and quantification of Cas12a-triggered disassembly of DNA tetrahedra.

We next tested the disassembly of the DNA tetrahedron using CRISPR-Cas12a-treated DNA or RNA invading strands with identical sequences. When the DNA invading strand was preincubated with active Cas12a, it was fully digested and therefore unable to initiate strand displacement. As a result, the tetrahedron remained intact (Figure 2d, lane 3). In contrast, the RNA invading strand was not affected by Cas12a, and upon addition to the tetrahedron, it successfully initiated strand displacement, leading to the complete disassembly of tetrahedron (Figure 2d, lane 5). DNA and RNA invading strands in the presence of inactive Cas12a (lanes 2 and 4) caused disassembly of the DNA tetrahedron (Figure 2e). To demonstrate that this reconfiguration is specifically due to the combination of Cas12a and the invading strand, we performed a similar experiment using DNA and RNA invading strands that do not correspond to the toehold. We observed that the tetrahedron did not disassemble in either case, indicating that an intact RNA does not cause disassembly unless it is a complement to the toehold (Figure S3). These results show that disassembly can be controlled selectively for DNA and RNA invading strands, introducing an additional level of regulation that operates independently of toehold design.

After demonstrating controlled disassembly, we investigated whether this strategy could be combined with target-specific regulation of DNA nanostructure reconfiguration. In the above experiments, Cas12a was activated with a crRNA designed against Target_L_ (crRNA_L_ for Target_L_). In contrast, incubation of the Cas12a-crRNA_L_ complex with genomic regions identified from *Campylobacter jejuni* and Shiga toxin-producing *Escherichia coli* (STEC), Target_J_ and Target_S_,^26^ did not activate Cas12a, thereby leaving the DNA invading strand intact and causing DNA tetrahedron disassembly (Figure S4). Based on this result, we extended this strategy to develop a multitarget responsive platform (Figure 3a). We used three genomic DNA targets (Target_L_, Target_J_, and Target_S_) and their corresponding crRNAs (crRNA_L_, crRNA_J_, and crRNA_S_) to study the response of different target combinations. When Cas12a was programmed with a single crRNA, only the genomic target complementary to the programmed crRNA activated the Cas12a. The activated Cas12a degrades the DNA invading strand, preventing strand displacement and keeping the tetrahedron intact. For the noncomplementary targets, Cas12a remained inactive, allowing the DNA invading strand to disassemble the tetrahedron (Figure 3b and Figure S4). Next, we examined whether the same strategy can be used for two genomic targets simultaneously when Cas12a was programmed with two crRNAs (Figure 3b and Figure S5). As expected, genomic targets complementary to either of the programmed crRNAs activated Cas12a and retained the intact tetrahedron, whereas the remaining off-target sequence led to strand displacement and tetrahedron disassembly. For example, the crRNA_L+J_ combination showed intact tetrahedron bands for Target_L_ and Target_J_, whereas the Target_S_ sample showed tetrahedron disassembly (Figure 3b). We observed similar results for the other crRNA combinations. Finally, we included all three crRNAs in the reaction mixture. Under these conditions, all three targets activated their corresponding crRNA-Cas12a complex, resulting in intact tetrahedron bands for Target_J_, Target_L_, and Target_S_ (Figure 3b and Figure S6). We observed that competition among the different crRNAs during Cas12a–crRNA complex formation may alter the relative abundance of the corresponding Cas12a–crRNA complexes, especially in the case of Cas12a–crRNA_J_, thereby reducing the effective amount of Cas12a–crRNA_J_ available for invading-strand cleavage when multiple crRNAs are present. Overall, an intact tetrahedron band was observed only when the programmed crRNA matched its corresponding genomic target, whereas the off-target sequences showed tetrahedron disassembly (Figure 3b, heat map). Together, these results demonstrate that our strategy can be readily adopted to respond to different combinations of genomic targets, allowing target-dependent regulation of DNA tetrahedron disassembly.

**Figure 3:**
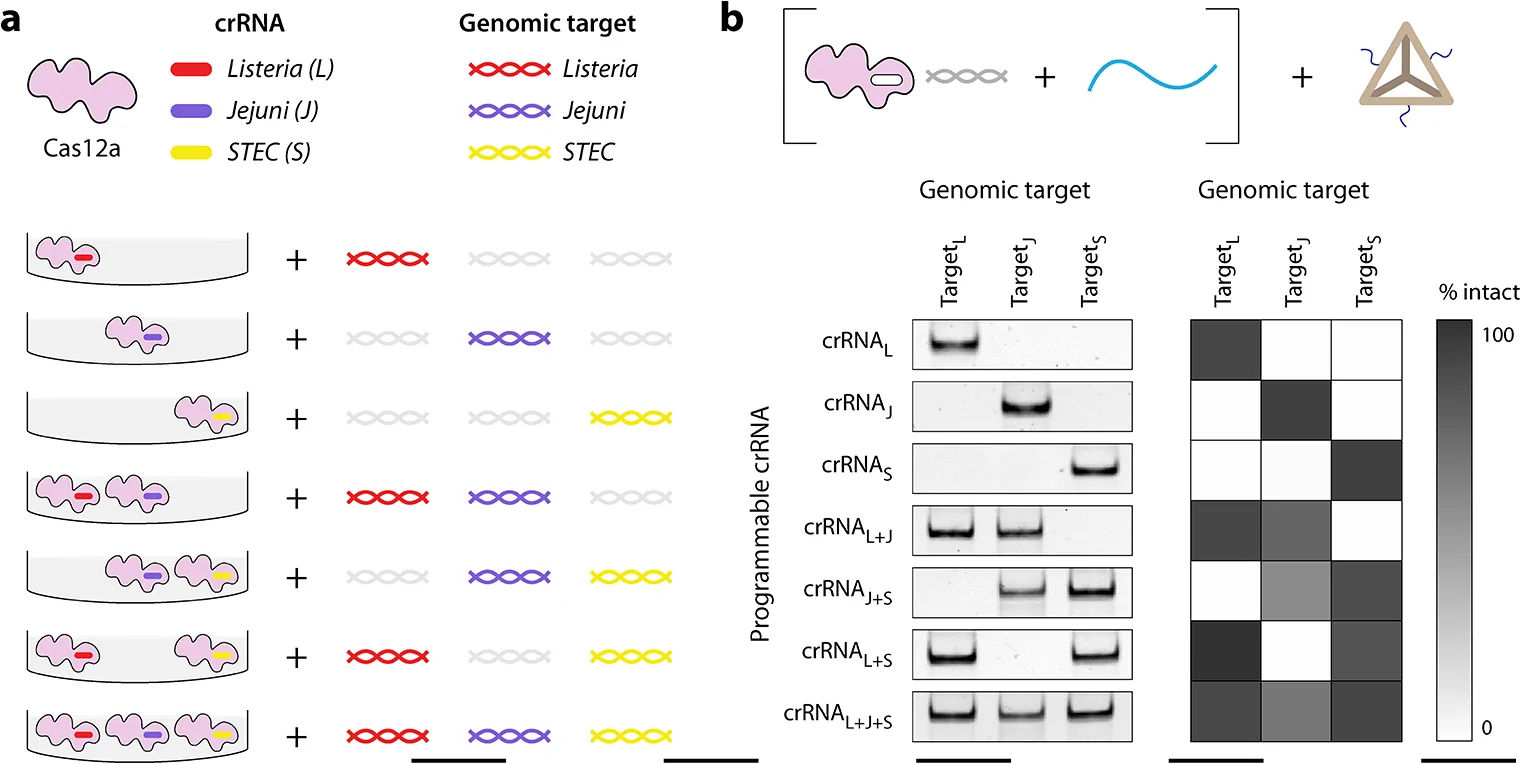
Target-specific regulation of DNA tetrahedron disassembly by CRISPR-Cas12a. (a) crRNA can be programmed for any of three genomic targets and is activated only in the presence of the correct pair of crRNA and genomic target. (b) Native PAGE analysis of the multitarget response using Target_J_, Target_L_, and Target_S_ with their corresponding crRNAs. The presence of a band indicates intact tetrahedra and absence indicates disassembled tetrahedra. Right: Heat map generated from the band intensities of gels shown on the left.

In this work, we developed an alternative approach to regulating DNA tetrahedron disassembly by controlling the availability of the invading strand. Unlike our previous strategy,^18^ where strand displacement was regulated by modifying the toehold, the tetrahedron structure remained unchanged in this study. Here, activated Cas12a selectively degraded the DNA invading strand, preventing strand displacement and maintaining the intact tetrahedron. We further showed that this response could be directly linked to genomic target recognition through programmable crRNAs. Only the complementary target activated Cas12a, while noncomplementary targets allowed strand displacement to proceed. The same strategy was successfully extended from single target response to different combinations of genomic targets by changing the crRNA composition. While our strategy involves a separate Cas12a treatment step for the invading strand, future studies could test treatment of the single stranded toehold regions on the tetrahedron. A one-pot reaction with Cas12a would react with the single stranded invading strand as well as the single stranded toehold on the tetrahedron and might interfere with input-based disassembly. Our results show that CRISPR-mediated target recognition can be combined with DNA strand displacement to produce a programmable structural response, adding to the existing CRISPR-based strategies to create functional DNA nanostructures.^27–30^ We believe this strategy can be adapted to other DNA nanostructures and may provide a useful platform for nucleic acid detection and stimuli-responsive DNA nanodevices.

## Supplementary Material



## Notes

Experimental procedures, additional experimental details, materials lists, and oligonucleotide sequences (PDF)
