# Supplementary material for: A CRISPR-Cas12a-Responsive DNA Tetrahedron
Source: Biochemistry. 2026 Sep 16;65(19):2935–9. doi: 10.1021/acs.biochem.6c00589 (PMC13648235; doi:10.1021/acs.biochem.6c00589)
Supplement: Supplementary file 1 [file bi6c00589_si_001.pdf]

Supporting information

A CRISPR-Cas12a-responsive DNA tetrahedron

Shubhajit Singha,[1] Mehmet V. Yigit,[1,2,*] and Arun Richard Chandrasekaran[2,3,*]

[1]Department of Chemistry, University at Albany, State University of New York, Albany, NY 12222, USA
[2]The RNA Institute, University at Albany, State University of New York, Albany, NY 12222, USA
[3]Department of Nanoscale Science and Engineering, University at Albany, State University of New York, Albany, NY 12222, USA.

Correspondence: myigit@albany.edu (MVY) or arun@albany.edu (ARC)

EXPERIMENTAL PROCEDURES.

Materials. All the DNA strands and crRNA constructs were obtained from Integrated DNA Technologies (IDT), Inc. (Table S1 and S2). EnGen Lba Cas12a (Cas12a) (catalog# M0653), and NEBuffer r2.1 10× reaction buffer (catalog# B6002S) were purchased from New England Biolabs.

Assembly of 3-point-star motif and the DNA tetrahedron. To assemble the 3-point-star motif, strands L, M, and S-blunt were mixed in a 1:3:3 molar ratio at a final DNA concentration of 100 nM in 1× TAE-Mg^{2+} buffer containing 40 mM Tris base (pH 8), 20 mM acetic acid, 2 mM EDTA, and 12.5 mM magnesium acetate. The mixture was annealed from 95 °C to 20 °C over 2 h using a Bio-Rad T100 thermal cycler. Strand S-blunt does not contain sticky ends and was used to prevent the assembly of higher order DNA nanostructures such as the DNA tetrahedron. To prepare the DNA tetrahedron with toeholds, strands L, M, and S-DNA-TH were mixed in a 1:3:3 molar ratio in 1× TAE-Mg^{2+} buffer at a final DNA concentration of 100 nM. Samples were placed in a beaker containing 2 liters of water heated to 95 °C, then placed in a Styrofoam box to cool to 20 °C over the course of 2 days.

Target identification and crRNA design. To identify a targetable region in *L. monocytogenes*, *C. jejuni*, and Shiga Toxin-producing *E. coli* (STEC) genomes ($Target_L$, $Target_J$, and $Target_S$), a conserved genomic region containing a canonical TTTN protospacer adjacent motif (PAM) and relatively high GC content were selected through sequence analysis using NCBI Nucleotide BLAST tool blasting against the core nucleotide database (core_nt) according to our earlier report (Hanson et al, *ACS Sens.* 2026, 11, 2774-2783). crRNAs corresponding to these sequences were designed using IDT website.

Cas12a-crRNA complex preparation and activation. Cas12a-crRNA complex was prepared at a 1:1.2 molar ratio of Cas12a to the corresponding crRNA (typical final concentrations of 1.0 µM Cas12a and 1.2 µM crRNA) in 1× NEBuffer r2.1, and the mixture was incubated at 37 °C for 30 minutes. To activate the Cas12a-crRNA complex the double stranded genomic target DNA was added at a final concentration of 12.5 nM. Based on the optimization experiments, a final Cas12a concentration of 125 nM was used throughout the study. The mixture was incubated at 37 °C for 45 min to activate Cas12a. After target incubation, the DNA or RNA invading strand was added to the Cas12a-target mixture and incubated at 37 °C for 1 h. The Cas12a-crRNA complex was then thermally deactivated by heating the mixture at 65 °C for 15 min.

DNA tetrahedron reconfiguration after CRISPR reaction and gel electrophoresis analysis. DNA tetrahedron sample (50 nM) was added to the Cas12a-treated invading strands (5-10-fold excess) and incubated at 20 °C for 1 h. Treated samples were run on a 4% non-denaturing polyacrylamide gel. Samples were mixed with 10× loading dye containing bromophenol blue and glycerol, then loaded in 4% non-denaturing polyacrylamide gels (19:1 acrylamide; National Diagnostics) prepared in 1× TAE-Mg^{2+} buffer. Electrophoresis was carried out at 4 °C using 1× TAE-Mg^{2+} as the running buffer. Following electrophoresis, gels were stained with 0.5× GelRed (Biotium) in deionized water for 20 min in the dark, then destained in deionized water for 10 min. Gels were imaged using a Bio-Rad Gel Doc XR+ imaging system with the standard default settings for GelRed with UV illumination. Gel images were analyzed using ImageLab software (Bio-Rad). Images were typically taken at multiple exposures. For replicate analysis, separate gels with the same exposure time were typically used to quantify band brightness. The DNA tetrahedron assembly band yield in each lane was normalized to the DNA tetrahedron control lane. The experiments were performed in triplicate, and the error bars were calculated by standard deviation (SD). Data are represented as Mean ± SD.

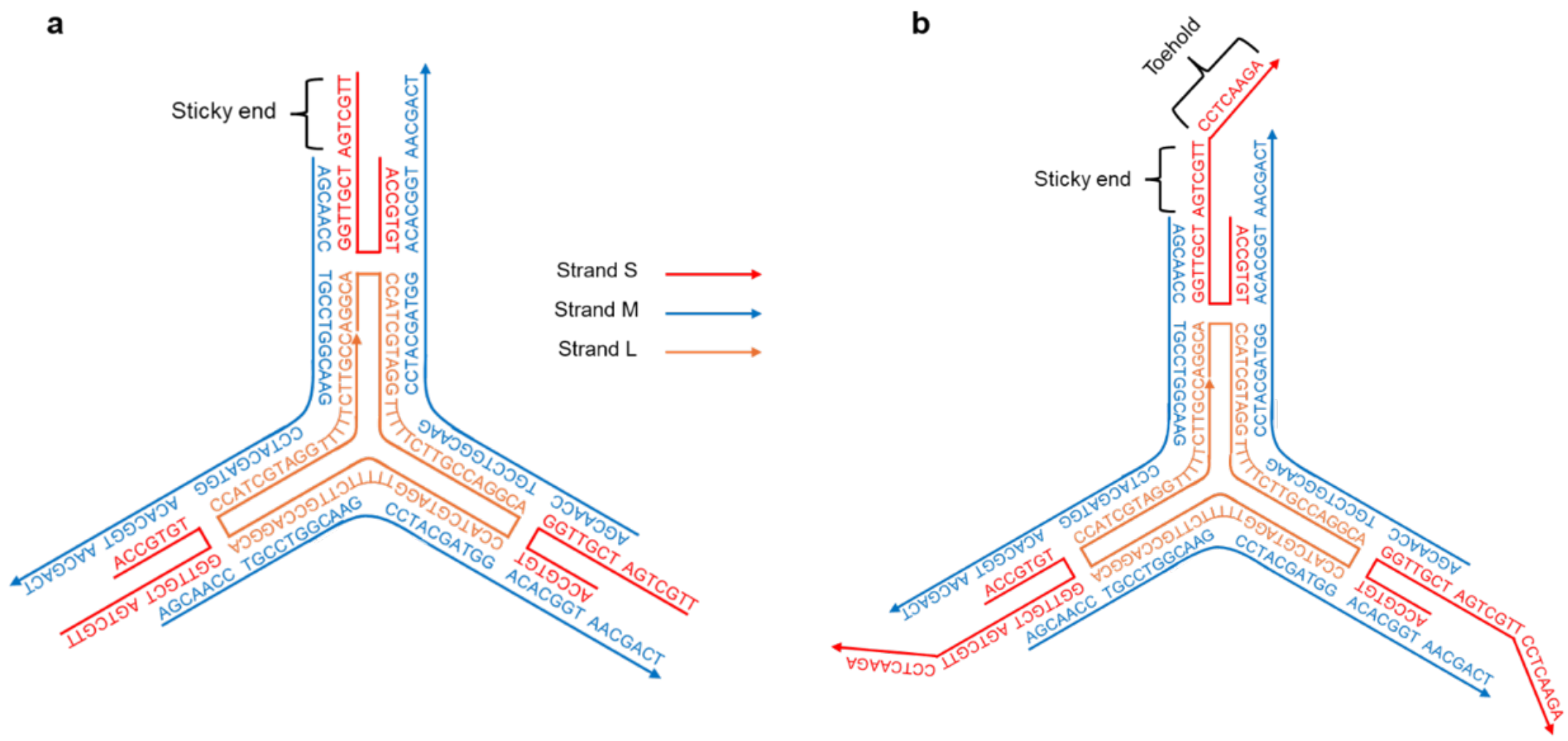

Figure S1. Sequence of the 3-point-star motif without toeholds (a) and with toeholds (b). The motif is three-fold symmetric and consists of strands L, M, and S in the ratio 1:3:3. Four such motifs connect via sticky end cohesion to assemble into the DNA tetrahedron. The additional single stranded extension in Strand S acts as the toehold for the strand displacement process.

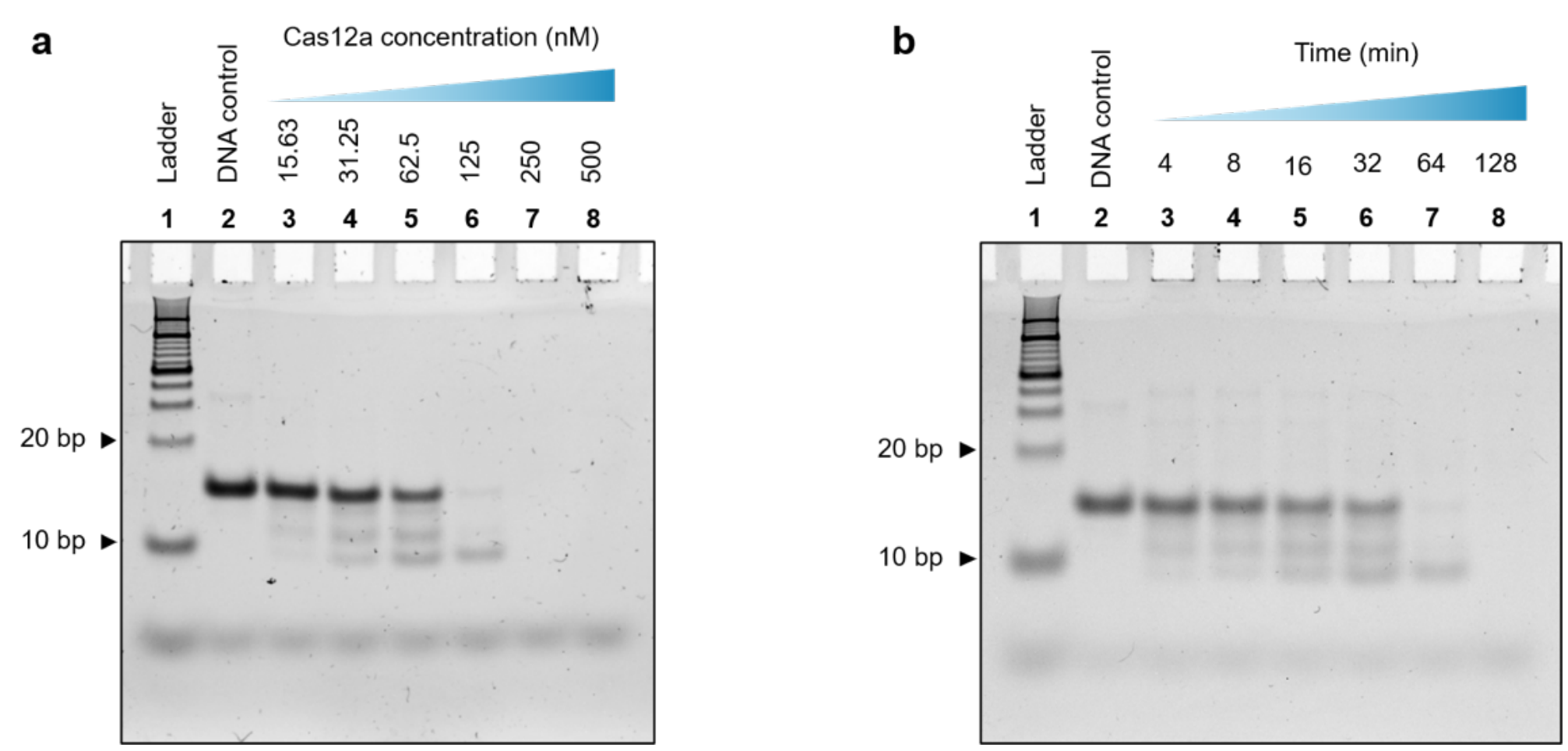

Figure S2. CRISPR-Cas12a reaction on the DNA invading strand. (a) Digestion of the DNA invading strand (1 µM) by different concentrations of activated Cas12a. (b) Time dependent digestion of the DNA invading strand (1 µM) by activated Cas12a (125 nM).

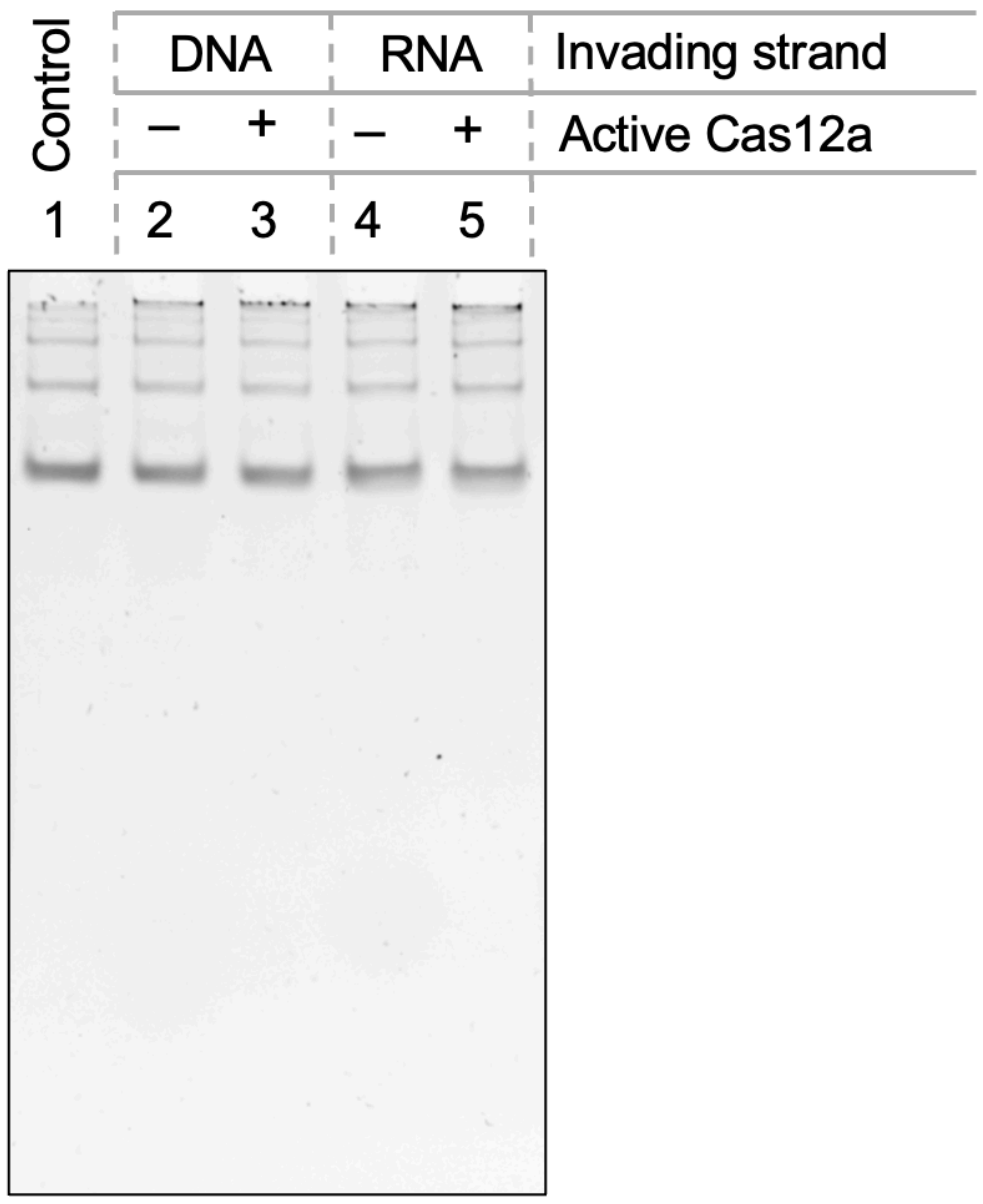

Figure S3. Testing DNA tetrahedron disassembly using invading strands with random sequences.

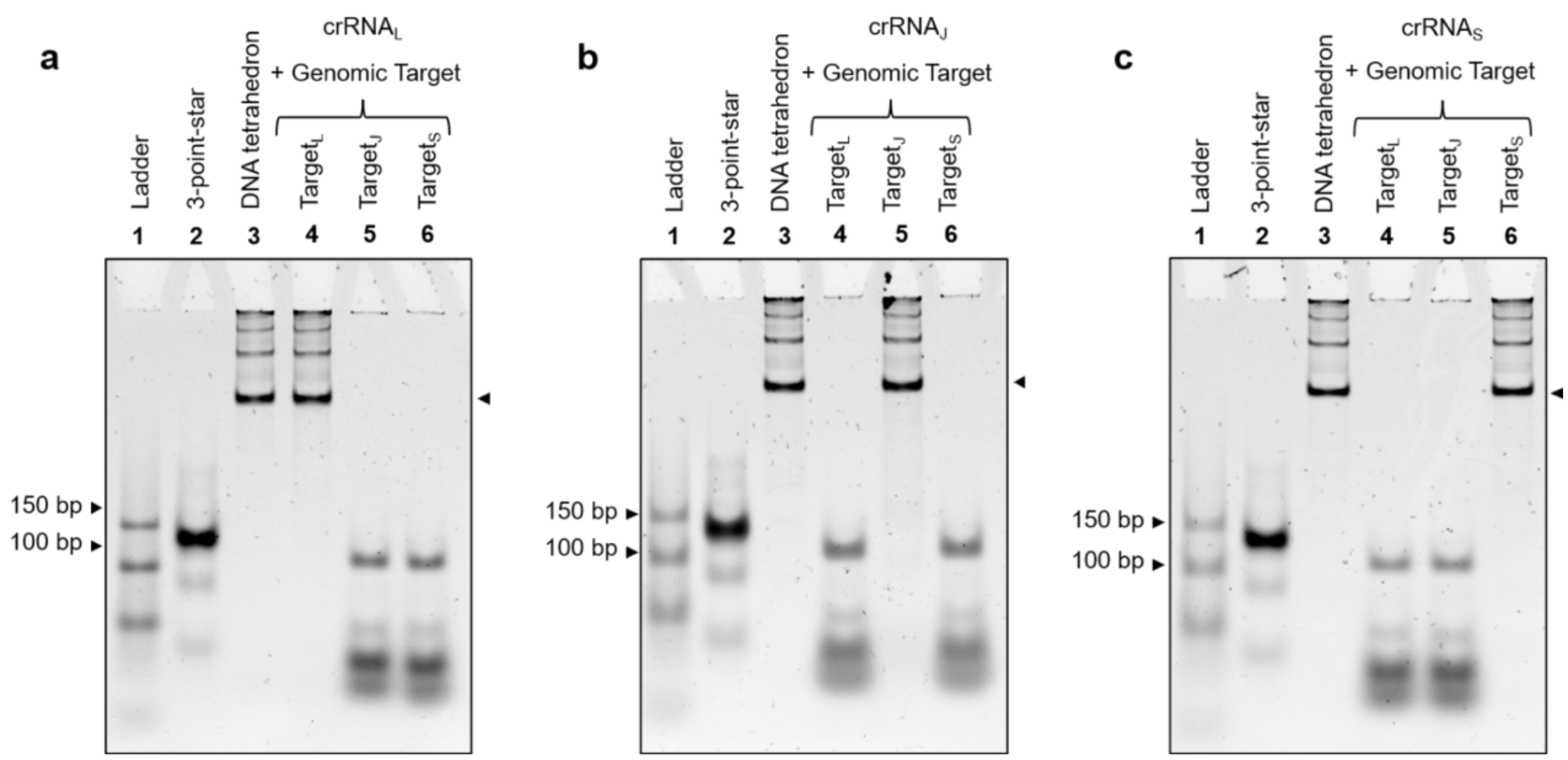

Figure S4. DNA tetrahedron disassembly using Cas12a programmed with one crRNA. The tetrahedron is disassembled only in the presence of the correct target dsDNA.

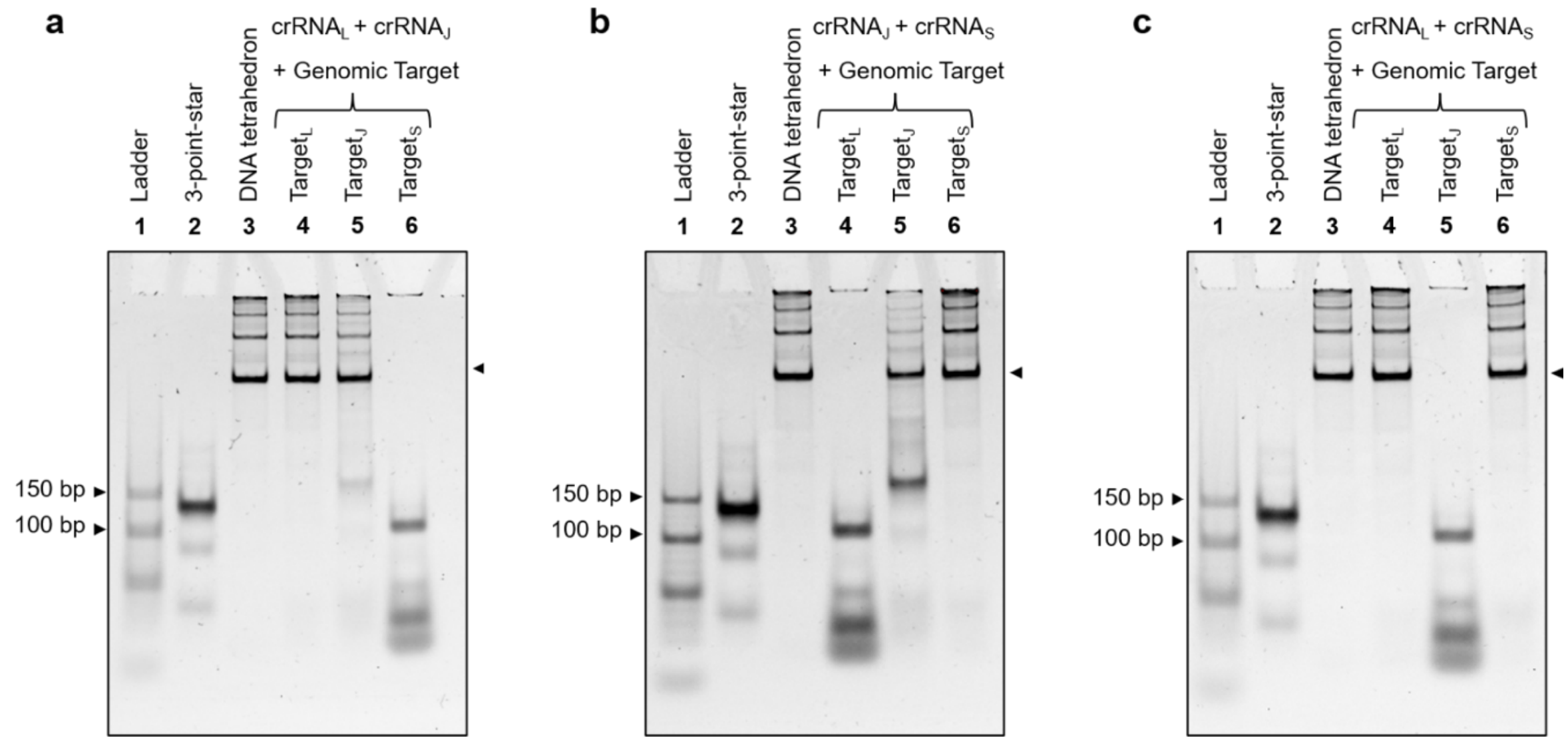

Figure S5. DNA tetrahedron disassembly using Cas12a programmed with two crRNAs. The tetrahedron is disassembled only in the presence of one of the two correct target dsDNAs.

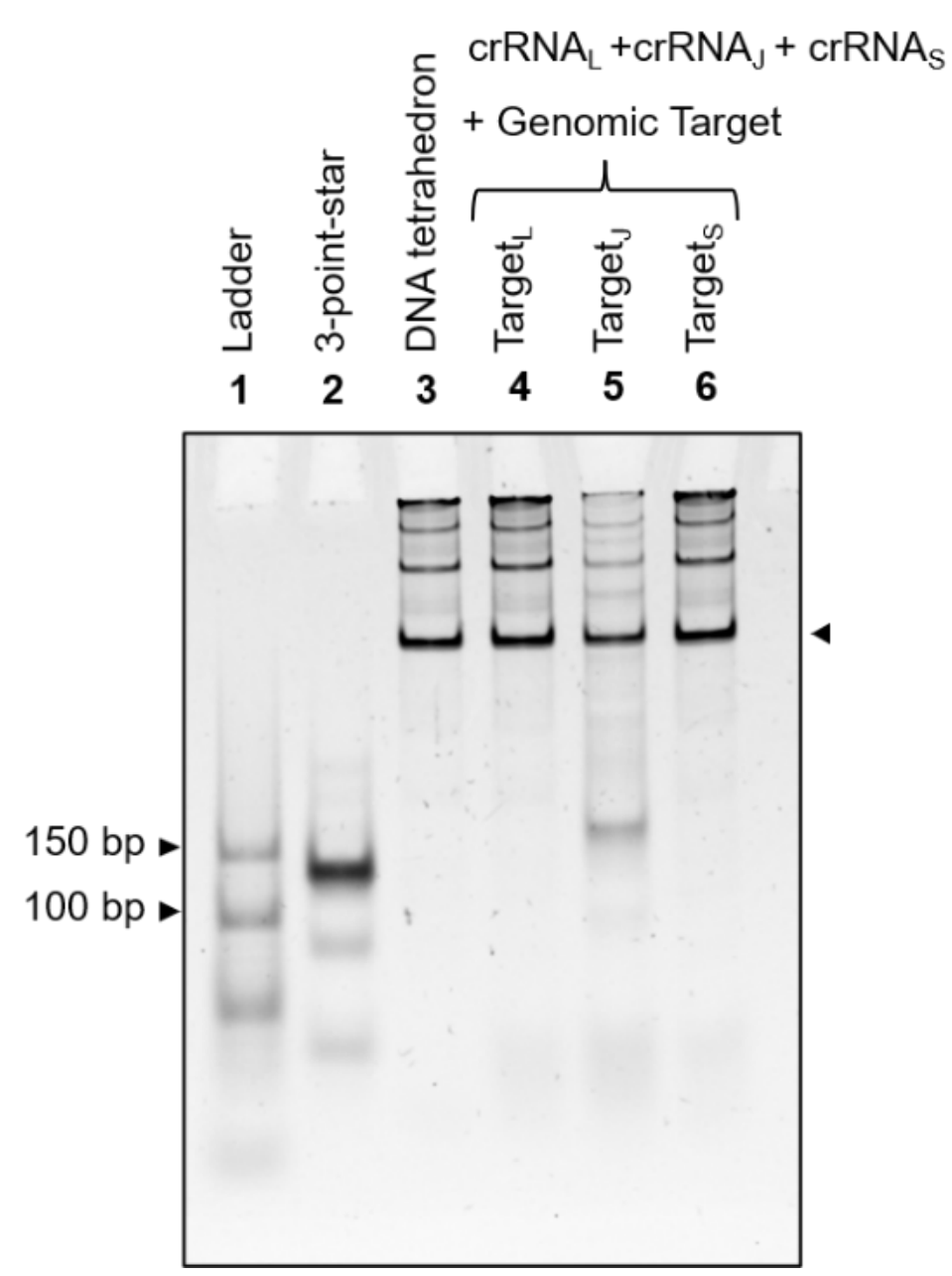

Figure S6. DNA tetrahedron disassembly using Cas12a programmed with three crRNAs. The tetrahedron is disassembled in the presence of any of the three target dsDNAs.

Name	Sequence (5' to 3')
Strand L	AGGCACCATCGTAGGTTTTTCTTGCCAGGCACCATCGTAGGTTTTTCTTGCCAG GCACCATCGTAGGTTTTTCTTGCC
Strand M	AGCAACCTGCCTGGCAAGCCTACGATGGACACGGT**AACGACT**
S-blunt	AGTCGTTACCGTGTGGTTGCT
S-DNA-TH	ACCGTGTGGTTGCT**AGTCGTT**CCTCAAGA
DNA invading strand	TCTTGAGGAACGACTAGCAACCACACGGT
RNA invading strand	UCUUGAGGAACGACUAGCAACCACACGGU

Table S1. Sequences of the tetrahedron, including invading strands. Sequences in bold are sticky ends, and underlined sequence is the toehold region.

<table>
<tr><th>Name</th><th>Sequence</th></tr>
<tr><td>crRNA_L</td><td>5' UAAUUUCUACUAAGUGUAGAUCGCUUACGGCAGCAUCAAAAGC 3'</td></tr>
<tr><td>crRNA_J</td><td>5' UAAUUUCUACUAAGUGUAGAUUGGAGCACUUCCAUGACCACCCC 3'</td></tr>
<tr><td>crRNA_S</td><td>5' UAAUUUCUACUAAGUGUAGAUCGAGUACAUUGGCAUCGUGUGG 3'</td></tr>
<tr><td>Target_L</td><td>5' AAAGTAAAAGCTGCTTTTGATGCTGCCGTAAGCGGAAAATCTGTCTCAGG 3'
||||||||||||||||||||||||||||||||||||||||||||||||||
3' TTTCATTTTCGACGAAAACTACGACGGCATTCGCCTTTTAGACAGAGTCC 5'</td></tr>
<tr><td>Target_J</td><td>5' AGTTATTGGAAGGGGTGGTCATGGAAGTGCTCCAGAAAAGGCAAAAGATC 3'
||||||||||||||||||||||||||||||||||||||||||||||||||
3' TCAATAACCTTCCCCACCAGTACCTTCACGAGGTCTTTTCCGTTTTCTAG 5'</td></tr>
<tr><td>Target_S</td><td>5' TTTTTACCCTGTCCACACGATGCCAATGTACTCGGAAAAATATCAAAAGC 3'
||||||||||||||||||||||||||||||||||||||||||||||||||
3' AAAAATGGGACAGGTGTGCTACGGTTACATGAGCCTTTTTATAGTTTTCG 5'</td></tr>
</table>

Table S2. List of crRNAs and corresponding truncated target sequences. For crRNAs, the target recognition region is bolded and underlined. For truncated target sequences, the recognition sites are bolded, and PAM sequences are bolded and underlined.
